# Effect of aging on acute pancreatitis through gut microbiota

**DOI:** 10.3389/fmicb.2022.897992

**Published:** 2022-07-28

**Authors:** Hui Jing, Qimeng Chang, Yayun Xu, Jianfa Wang, Xubo Wu, Jiating Huang, Lishun Wang, Ziping Zhang

**Affiliations:** ^1^Department of Hepatopancreatobiliary Surgery, Minhang Hospital, Fudan University, Shanghai, China; ^2^Institute of Fudan-Minhang Academic Health System, Minhang Hospital, Fudan University, Shanghai, China; ^3^Center for Traditional Chinese Medicine and Gut Microbiota, Minhang Hospital, Fudan University, Shanghai, China

**Keywords:** acute pancreatitis, aging, antimicrobial peptides, bacterial translocation, gut microbiota

## Abstract

**Background:**

Compared to younger people, older people have a higher risk and poorer prognosis of acute pancreatitis, but the effect of gut microbiota on acute pancreatitis is still unknown. We aim to investigate the effect of aging gut microbiota on acute pancreatitis and explore the potential mechanism of this phenomenon.

**Methods:**

Eighteen fecal samples from healthy adult participants, including nine older and nine younger adults were collected. C57BL/6 mice were treated with antibiotics for fecal microbiota transplantation from older and younger participants. Acute pancreatitis was induced by cerulein and lipopolysaccharide in these mice. The effect of the aged gut microbiota was further tested *via* antibiotic treatment before or after acute pancreatitis induction.

**Results:**

The gut microbiota of older and younger adults differed greatly. Aged gut microbiota exacerbated acute pancreatitis during both the early and recovery stages. At the same time, the mRNA expression of multiple antimicrobial peptides in the pancreas and ileum declined in the older group. Antibiotic treatment before acute pancreatitis could remove the effect of aging gut microbiota, but antibiotic treatment after acute pancreatitis could not.

**Conclusion:**

Aging can affect acute pancreatitis through gut microbiota which characterizes the deletion of multiple types of non-dominant species. This change in gut microbiota may potentially regulate antimicrobial peptides in the early and recovery stages. The level of antimicrobial peptides has negative correlations with a more severe phenotype.

## Introduction

Acute pancreatitis (AP) has a global incidence of 33.74 cases per 100,000 person-years (Xiao et al., 2016). The risk of AP increases rapidly with age in both men and women (Yadav and Lowenfels, 2013; Sellers et al., 2018; Petrov and Yadav, 2019). Older patients with AP have lower short-and long-term survival rates (Husu et al., 2019) and a higher risk of organ failure (Schepers et al., 2019). Infection in both pancreatic and peripancreatic tissue are considered caused by translocation of bacteria in intestinal (Fritz et al., 2010). Gut microbiota significantly changes with age (Lynch and Pedersen, 2016). Recent studies have used the gut microbiota of elderly humans and children to investigate its role in osteoporosis (Liu et al., 2021). Gut microbiota also closely related to AP (Zhu et al., 2019; Li et al., 2020; Yu et al., 2020; van den Berg et al., 2021). While there are few works testified that aging worsens the acute pancreatitis of animals (Miyasaka et al., 1992; Fu et al., 2012; Coelho et al., 2019). Coelho etc. find that aged rats appear more severe acute pancreatitis and increased bacterial translocation than young rats (Coelho et al., 2019). However, whether aging affects acute pancreatitis through gut microbiota is still unknown. Antimicrobial peptides (AMPs) that are also affected by aging contribute to innate immunity by targeting negatively charged membranes of microbes (Branca et al., 2019; Hanson et al., 2021). There is only few works about AMPs in acute pancreatitis(Huang et al., 2017), but there are great studies on the effect of antimicrobial peptides in lethal bacteremia nowadays (Ahuja et al., 2017; Ho et al., 2020). This suggests that AMPs may be related to the second infection of severe acute pancreatitis. Also, whether aging gut microbiota affects the secretion of AMPs in acute pancreatitis is still unknown.

In this study, we hypothesize that aged gut microbiota contributes to AP. We aim to explore the relationship between the aged gut microbiota and AMPs secretion and the effect of aging on AP and investigate the impact of the timing of the antibiotic intervention on the changes caused by aging gut microbiota. Faeces of people of different ages were collected. By analyzing the results of 16SrDNA sequencing, we derived a pattern of aging in the gut microbiota of the elderly characterized by a decrease in non-dominant flora. Then, we constructed a mouse model of humanized gut microbiota to explore that aging gut microbiota could aggravate the phenotype of acute pancreatitis in both early-stage and recovery stage. We observed pancreatic and ileum AMPs secretion levels and analyzed their correlation with disease phenotypes. Then, antibiotics were cleared before the induction of the disease, and the differences in phenotype and antimicrobial peptide secretion were determined to be derived from the composition of gut microbiota at the onset of the disease. Finally, we conducted antibiotic intervention after disease induction to simulate clinical treatment and found that antibiotic intervention could not completely improve the negative effects of gut microbiota of the elderly. Our study firstly confirms the relationship between aged gut microbiota and acute pancreatitis and provides a potential mechanism for the phenomenon. The findings will provide new insights for future clinical studies and treatment targets.

## Materials and methods

### Participants and sample collection

Eighteen healthy adults were included in this study. The donors of the fresh stool samples included younger (age > 15 and < 45 years, *n* = 9) and older individuals (age > 65 years, *n* = 9). Donors would be included if they fit all following conditions: (1) no history of use of antibiotics or probiotics for at least 3 months; (2) in good physical health and mental health, not undergoing medical treatment; (3) no history of severe disease of the digestive tract; (4) no history of major operation of the digestive tract; (4) no history of faecal microbiota transplantation treatment; (5) no special dietary habits. Fresh stool samples from the donors were collected and immediately transported to the laboratory at 4°C. The samples were homogenized with 30% glycerol at a 1:1 (w/v) ratio, split into 50 ml centrifuge tubes, and frozen at −80°C for long-term storage. The study design was approved by the Ethics Committee of Minhang Hospital, Fudan University, and informed consent was obtained from all participants.

### Mice

Six-week to eight-week-old male C57BL/6 mice were purchased from Charles River Laboratories (Hangzhou, China) and housed under standard conditions (12 h dark/light cycle) with free access to standard chow and water. After 1 week of adaptation, the mice were used in the experiments. Blood was drawn *via* the inferior vena cava, followed by the aseptic removal of the biggest mesenteric lymph node and pancreas and other contents. Aliquots were fixed in 4% paraformaldehyde or stored at −80°C. FMT was performed to build humanized gut microbiota mice model. Briefly, C57BL/6 mice were orally gavaged with broad-spectrum cocktail antibiotics(ABX) once a day for 5 days. The ABX contains vancomycin (100 mg/kg), neomycin sulfate (200 mg/kg), metronidazole (200 mg/kg), and ampicillin (200 mg/kg) to remove indigenous gut microbiota. Fecal samples were collected and plated under aerobic condition with Columbia blood medium, brain heart infusion medium and Man Rogosa Sharpe medium, respectively, and anaerobic conditions with anaerobe agar to confirm microbiota depletion. Also, general PCR was performed with primers of 16 s regine and reaction products were visualized by agarose gel electrophoresis to confirm microbiota depletion. After antibiotic treatment, stool samples were filtered and washed twice with phosphate-buffered saline (PBS), resuspended in PBS at a ratio of 1 g/5 ml, and then orally gavaged to mice at a volume of 200 μl. FMT was performed serially for the first 3 days and then twice a week for 3 weeks. The recipient mice were housed in separate cages according to the different doner, with no more than 5 mice in each cage. The experiment of acute pancreatitis after FMT was repeated twice independently. Mice was housed separately by different doner, with one human donor corresponds to four or five recipient mice. We used a total of 4 young donors and 4 old donors in key experiments for 4–5 recipient mice per human donor. In the experiment of antibiotic treatment in the acute stage and antibiotic treatment in the recovery stage, we selected one representative donor of each group for FMT modeling. All animal studies were approved by the Ethics Committee of Minhang Hospital, Fudan University.

### Mouse model of severe acute pancreatitis

We constructed an AP model using a previously described method (Ding et al., 2003). Briefly, mice were challenged with 10 hourly intraperitoneal injections of cerulein (Sigma-Aldrich, St. Louis, Missouri, United States, 50 μg/kg, solubilized in PBS at a final concentration of 15 μg/ml), and intraperitoneal injection of one dose of lipopolysaccharide immediately after last shot of cerulein(Sigma-Aldrich, St. Louis, Missouri, United States; 10 mg/kg).

### Bacterial dissemination

Fresh blood and mesenteric lymph nodes were collected under non-anaerobic aseptic conditions and immediately placed in a sealed EP tube after tissue removal. The freshly removed tissue was transferred to an anaerobic condition within 15 min. Tissue grinding, dilution and inoculation were performed in an anaerobic condition. Full blood was 20-fold serial diluted with PBS. The largest mesenteric lymph nodes were homogenized in PBS and serially diluted 10-fold. The suspension was plated on anaerobic agar under strictly anaerobic conditions (80% N_2_, 10% H_2_, and 10% CO_2_) at 37°C in an anaerobic chamber. CFU was counted after 48 h of incubation, then normalized by volume (blood) or weight (mesenteric lymph nodes).

### Amplicon sequencing of 16S rRNA gene

Stool samples were snap-frozen in liquid nitrogen and stored at −80°C until use. Genomic DNA was extracted using the cetyltrimethylammonium bromide method. DNA purity was evaluated *via* electrophoresis on a 1% agarose gel, and the concentration was determined *via* UV spectrophotometry. The sample was diluted to 1 ng/μL with sterile water. Using diluted genomic DNA as a template, 16S rRNA gene amplicons targeting the V4 region were generated. The primer sequences were as follows: 515F (5’-GTGCCAGCMGCCGCGGTAA-3′) and 806R (5’-GGACTACHVGGGTWTCTAAT-3′). High-Fidelity PCR Master Mix (New England Biolabs Phusion^®^) with GC buffer and high-fidelity enzyme was used for polymerase chain reaction (PCR) amplification to ensure efficiency and accuracy. The PCR products were detected *via* electrophoresis on a 2% agarose gel. The qualified PCR products were purified using magnetic beads, quantified by enzyme standard, and mixed in equal quantities according to the concentration of PCR products, which were detected *via* 2% agarose gel electrophoresis. The target bands were recovered using gel recovery kits provided by Qiagen. A TruSeq^®^ DNA PCR-free sample preparation kit was used for library construction. The constructed library was quantified using Qubit and Q-PCR, and then NovaSeq6000 was used for machine sequencing.

### Microbiome bioinformatic analysis

Alpha diversity of gut microbiota was analyzed using QIIME2 (2019.4) software according to ASV information. Alpha diversity includes observed species, Chao 1 index, pielou_e index, Shannon index, Simpson index, goods coverage and PD whole tree. Beta diversity was estimated using weighted and unweighted UniFrac metrics between the samples. PCoA was performed in R software. We took the stool samples of mice after FMT as the sink, and the corresponding human feces of the donor and the stool samples of five mice without any treatment as the sources for SourceTracker analysis. Differential abundances at the genus level were identified using the R package DESeq2. The R package, CORRPLOT, was used to determine the correlation index.

### Measurement of plasma

Serum amylase activity was measured using an enzyme assay kit (Nanjing Jiancheng, C016-1-1, China). The levels of IL-6 in the plasma were mesureed with ELISA kits (Multi Sciences, EK206/3–96, China; intra-assay CV 2.9%). The levels of CRP in the plasma were mesureed with ELISA kits (Multi Sciences, EK294/3–96, China; intra-assay CV 1.4%). Experiment below was performed in duplicates following the manufacturer’s instructions.

### Histology

Pancreatic head tissues were fixed in 4% paraformaldehyde for at least 24 h. The pancreatic heads were stained with hematoxylin and eosin, examined, and scored with a light microscope. We selected 5 visual fields for each sample under pathological 20x magnification and the histological score was calculated by interstitial oedema, inflammatory infiltration, parenchymal necrosis, and parenchyma hemorrhage (Shimizu et al., 2000).

### RNA isolation and quantitative reverse transcription PCR

Tissues were collected under aseptic conditions and stored in aliquots after quick-freezing in liquid nitrogen at −80°C. To ensure aseptic conditions, 20–30 mg tissue was homogenised in a 1.5 ml ep tube using a disposable tissue grinding pestle in liquid nitrogen. Total RNA was isolated using a Tissue RNA Purification Kit (ES Science, RN002plus), according to the manufacturer’s instructions. RNA (500 ng) was reverse-transcribed using the PrimeScriptTM RT reagent Kit with gDNA Eraser (TaKaRa, RR047A), and quantitative PCR was performed using PowerUpTM SYBRTM Green Master Mix (TaKaRa) on a QuantStudio 7 Flex (Thermo Fisher). The primers were shown in Supplementary Table S1.

### Statistical analysis

Data were analyzed using either R (version 4.1.2) or GraphPad Prism 9. Statistical analysis was conducted using Student’s t-test, Fisher’s exact test, and Mann–Whitney test. Statistical significance was set at *p* < 0.05. The significance levels are indicated as follows: ^*^p < 0.05, ^**^p < 0.01, ^***^p < 0.001, ^****^p < 0.0001; ns, no significant difference.

## Results

### Gut microbiota between healthy older and younger people show great differences in bacterial diversity

**Figure 1:**
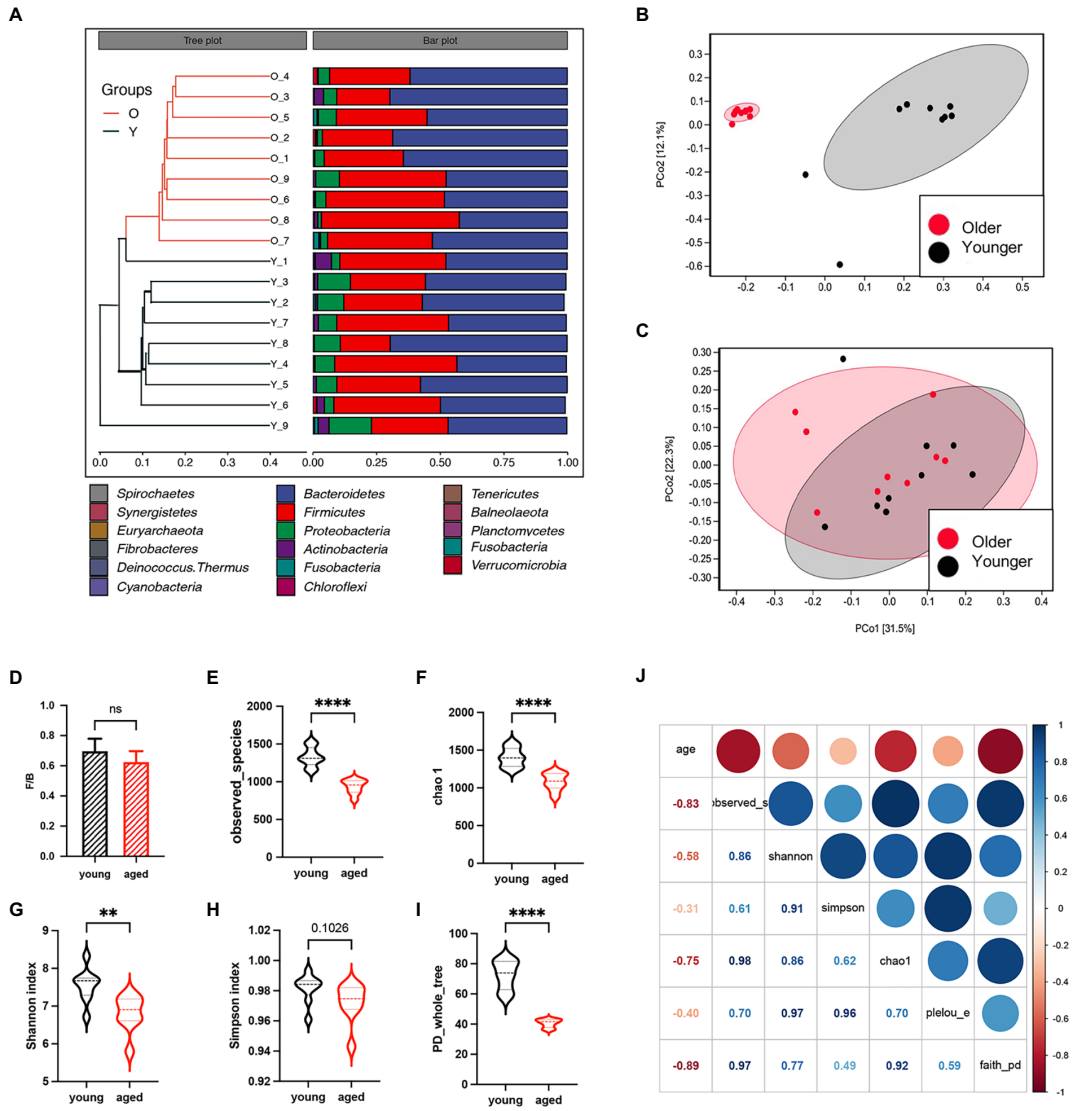
Gut microbiota between healthy older and younger people. **(A)** Unweighted pair group method with arithmetic mean clustering of samples shows younger and older groups were separated in the phylogenetic tree. **(B)** Principal coordinates analysis based on weighted UniFrac metrics. **(C)** Principal coordinates analysis based on unweighted UniFrac metrics. **(D)** The Firmicutes/Bacteroidota ratio of the two groups. **(E–I)** Alpha diversity indicators of the two groups. **(J)** The heatmap of correlation between alpha diversity and age.

Eighteen healthy adults were enrolled in this study. Sex was comparable between the healthy young group and healthy aged group (six females in each group). The unweighted pair group method with arithmetic mean clustering of samples showed that younger and older groups were separated in the phylogenetic tree, and the relative abundance of microbiota other than the two major phyla (Bacteroidota and Firmicutes) decreased (Figure 1A). Interestingly, the results of PcoA based on weighted UniFrac metrics showed no separation between the two groups (Figure 1B), whereas that based on unweighted UniFrac metrics showed a great distance between the two groups (Figure 1C), suggesting that the difference between the two groups was caused by bacterial diversity. No significant difference in the Firmicutes/Bacteroidota ratios of the two groups (Figure 1D) indicates that neither group had gut microbiota disorders. Multiple alpha diversity indicators showed significant differences between the two groups (Figures 1E–I). The heatmap in Figure 1J indicates strong correlations between age and alpha diversity of the gut microbiota.

### Gut microbiota of older people shows deletion of multiple types of non-dominant species

**Figure 2:**
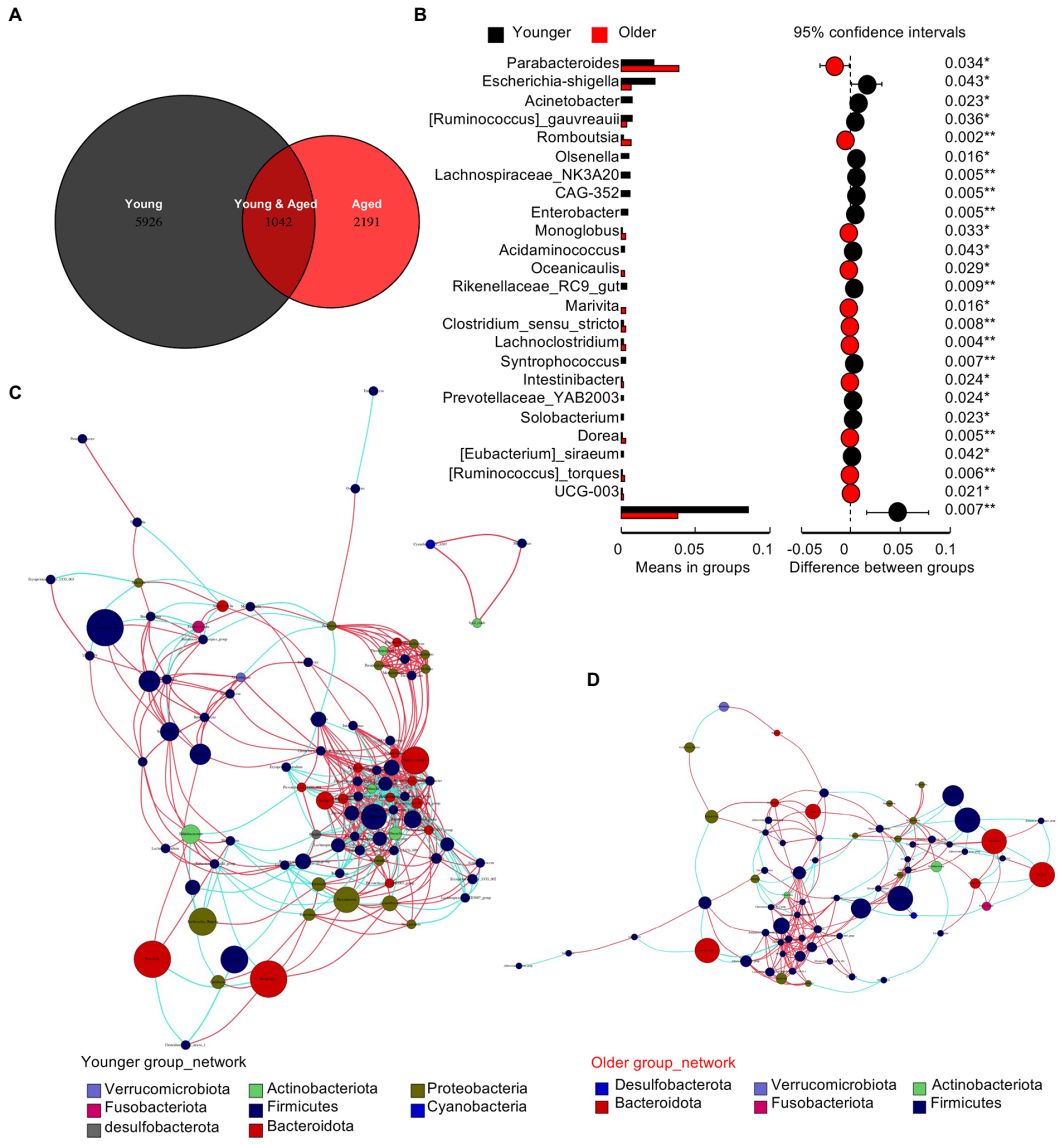
Gut microbiota of older people show the deletion of multiple types of non-dominant species. **(A)** Venn diagram of the two groups. **(B)** The *t* test between the two groups at the genus level. **(C)** Co-occurrence network diagrams of the healthy young group. **(D)** Co-occurrence network diagrams of the healthy older group.

The Venn diagram in Figure 2A shows a drastic decrease in specific operational taxonomic units (OTUs) in the healthy older group. To identify the different species between the two groups, we performed a t-test at the genus level (Figure 2B). The results showed that the differences were concentrated in the deletion of non-dominant species in the older group. Co-occurrence network diagrams of the healthy younger (Figure 2C) and older (Figure 2D) groups indicate a closer and more complex relationship in the gut microbiota of young people and a more intricate regulatory relationship between the gut microbiota and human body.

### Gut microbiota from healthy older people exacerbates the severity of sap in mice in the early stage

**Figure 3:**
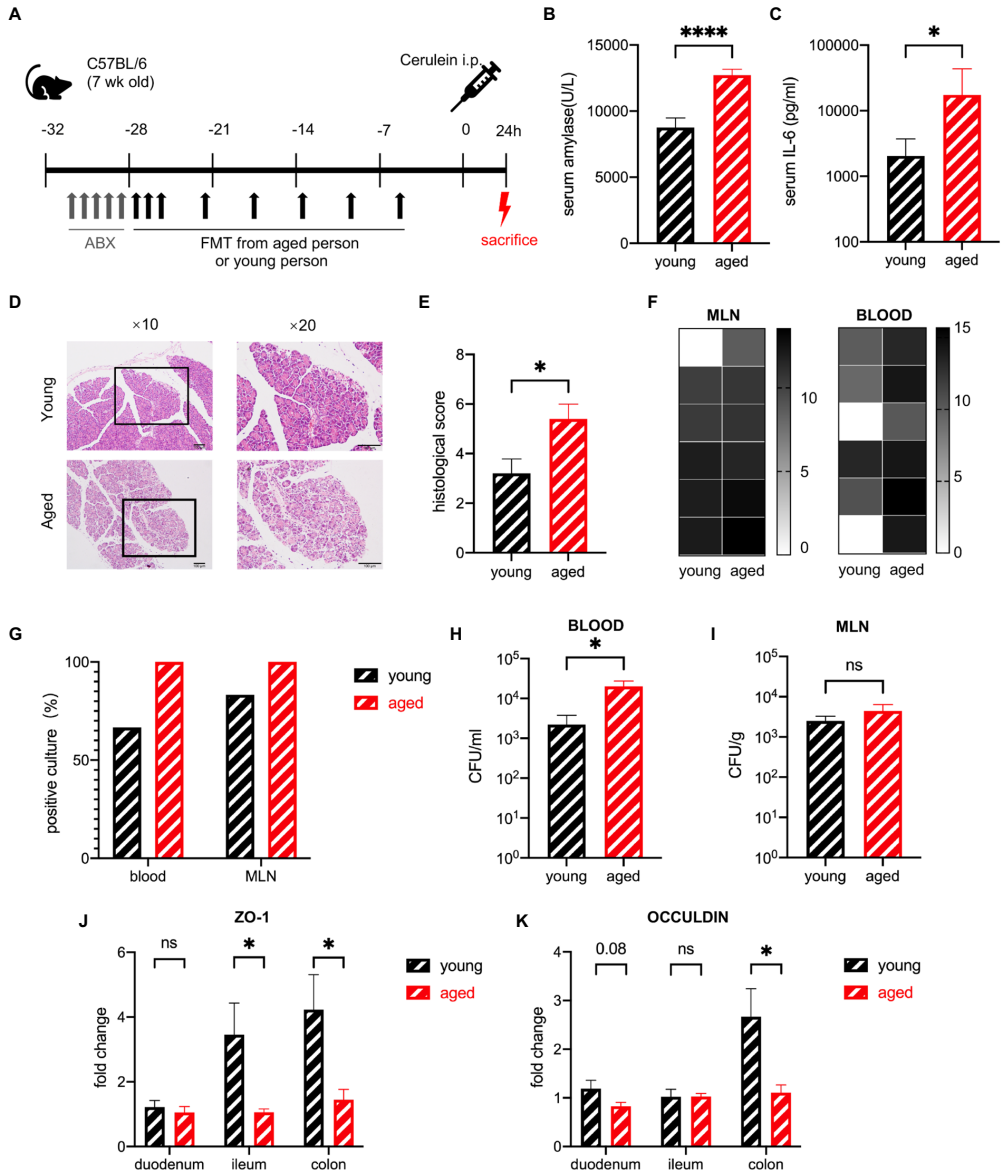
Gut microbiota of healthy older people increase the severity of SAP in mice in the early stage. **(A)** Mouse model of severe acute pancreatitis. **(B)** Serum amylase levels are significantly increased in the old group (*n* = 19) compared to the young group (*n* = 16). **(C)** Serum IL-6 levels are significantly increased in the old group (*n* = 14) compared to the young group (*n* = 12). **(D)** Representative hematoxylin and eosin staining of pancreatic tissue. **(E)** Histopathological scoring of pancreatic damage (*n* = 5 per group). **(F)** Heatmap of the CFU (log2) of blood and MLN (*n* = 6 per group). **(G)** Frequency of culture positivity in blood and MLN (*n* = 6 per group). CFU (*n* = 6 per group) in the blood **(H)** and MLN **(I)**. **(J)** Expression of tight junction genes ZO-1 in the duodenum, ileum, and colon of the young group (*n* = 5) versus the old group (*n* = 5). **(K)** Expression of tight junction genes OCCULDIN in the duodenum, ileum, and colon of the young group (*n* = 5) versus the old group (*n* = 5). ^*^p < 0.05, ^**^p < 0.01, ^***^p < 0.001, ^****^p < 0.0001, ns, no significant difference.

To investigate whether the aged gut microbiota affects the severity of AP, mice were treated with FMT, which was created from healthy human stool, for 3 weeks, after a five-day broad-spectrum antibiotic cocktail treatment. The efficiency of ABX was tested by general PCR of mice stool samples (supplementary Figure S2A). The efficiency of FMT was tested by SourceTracker (Supplementary Figure S2B). Severe acute pancreatitis (SAP) was induced by intraperitoneal injection of 10 shots of cerulein and one shot of lipopolysaccharide. The mice were sacrificed 24 h after the first injection (Figure 3A). SAP developed in both groups of mice treated with samples from older and younger participants (hereafter, for convenience, these two groups of mice will be called “old group” and “young group,” respectively) as confirmed by the elevated serum amylase levels. The serum levels of amylase and interleukin-6 (IL-6) were significantly higher in the old group than in the young group (Figures 3B,C). Histological examination showed that pancreatic injury characterized by interstitial oedema, inflammatory cell infiltration, and necrotic acinar cells were heavier in the old group, and the quantitative index showed a significant difference between the two groups (Figures 3D,E). To explore whether this difference was due to intestinal bacterial translocation, blood and homogenized mesenteric lymph nodes (MLNs) were cultured on anaerobic agar plates under strictly anaerobic conditions to ensure that both anaerobic bacteria and facultative anaerobic bacteria can grow. The abundance of translocated bacteria (calculated by colony forming units (CFU)/ml blood and CFU/g MLN) and the frequency of positive cultures showed a more severe bacterial translocation in the old group; blood CFUs were significantly higher in the old group (Figures 3F–I). Decreased gene expression of tight junction proteins, which indicates increased gut barrier permeability, supported the evidence of intestinal bacterial translocation. The most significant changes in gut barrier permeability occurred in the colon (Figures 3J,K).

### Gut microbiota from healthy older people delays the recovery from sap in mice

**Figure 4:**
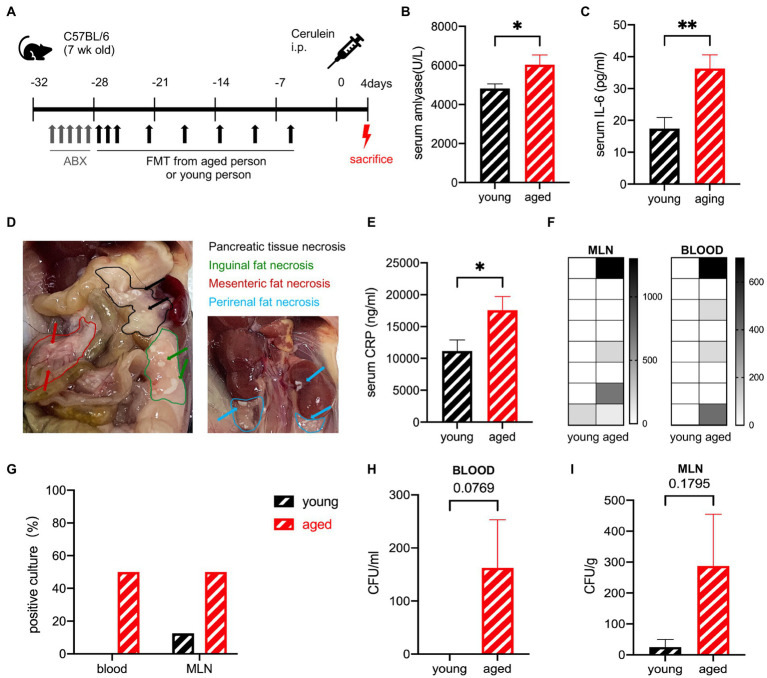
**(A)** Mouse model of severe acute pancreatitis. **(B)** Serum amylase levels are significantly increased in the old group (*n* = 9) compared to the young group (*n* = 10). **(C)** Serum IL-6 levels are significantly increased in the old group (*n* = 8) compared to the young group (*n* = 8). **(D)** Pancreatic and extra pancreatic fat necrosis in mice in the old group on day 4. **(E)** Serum CRP levels are significantly increased in the old group (*n* = 8) compared to the young group (*n* = 8). **(F)** Heatmap of the CFU(log2) of blood and MLN (*n* = 8). **(G)** Frequency of culture positivity in blood and MLN (*n* = 8 per group). CFU (*n* = 8 per group) in the blood **(H)** and MLN **(I)**. ^*^p < 0.05, ^**^p < 0.01, ^***^p < 0.001, ^****^p < 0.0001, ns, no significant difference.

The effects of the aged gut microbiota on the ability to recover from SAP were detected in the same experiment, where the mice were sacrificed after 4 days (Figure 4A). The serum levels of amylase and IL-6 were still significantly higher in the old group than in the young group but decreased rapidly in 24 h (Figures 4B,C). Pancreatic tissue necrosis and fat necrosis were observed in multiple organs of mice in the old group (Figure 4D). No visible necrosis was observed in the young group (Supplementary Figure S3A). Pathological examination showed that oedema and inflammatory cell infiltration had basically recovered, but necrosis was observed in some individuals in the older group (Supplementary Figure S3B). The serum C-reactive protein (CRP) level was also significantly higher in the old group (Figure 4E). The recovery of the old group was delayed compared to that of the young group. To detect bacterial translocation, we performed a bacterial culture. The young group had no positive blood culture, but the old group still had positive cultures at a ratio of 50%, suggesting that transient bacteremia occurred in the young group and persistent bacteremia was more likely to occur in the old group. The CFUs in both the blood and MLN were higher in the old group (Figures 4F–I).

### The expression of multiple antimicrobial peptides in the pancreas and ileum declined in the older group

**Figure 5:**
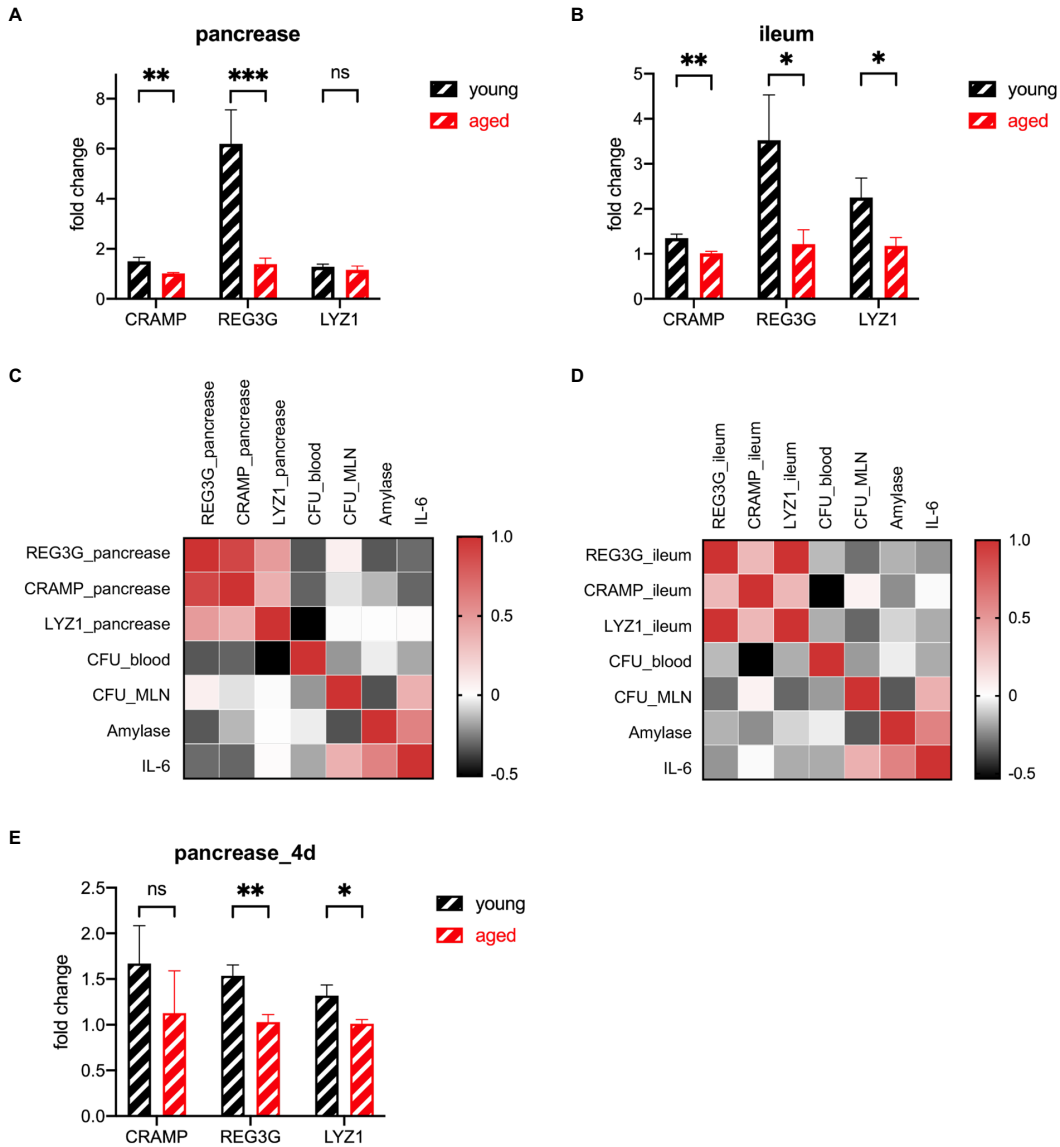
Expression of AMP genes in the pancreas **(A)** and ileum **(B)** of the young group (*n* = 5) versus the old group (*n* = 5) at the lethal endpoint of 24 h. **(C)** Heatmap of the Spearman correlation coefficients between the AMPs of the pancreas and other indicators. **(D)** Heatmap of the Spearman correlation coefficients between the AMPs of the ileum and other indicators. **(E)** Expression of AMP genes of the pancreatic tissue of the young group (*n* = 5) versus the old group (*n* = 5) at the lethal timepoint of 4 d. ^*^p < 0.05, ^**^p < 0.01, ^***^p < 0.001, ^****^p < 0.0001, ns, no significant difference.

**Figure 6:**
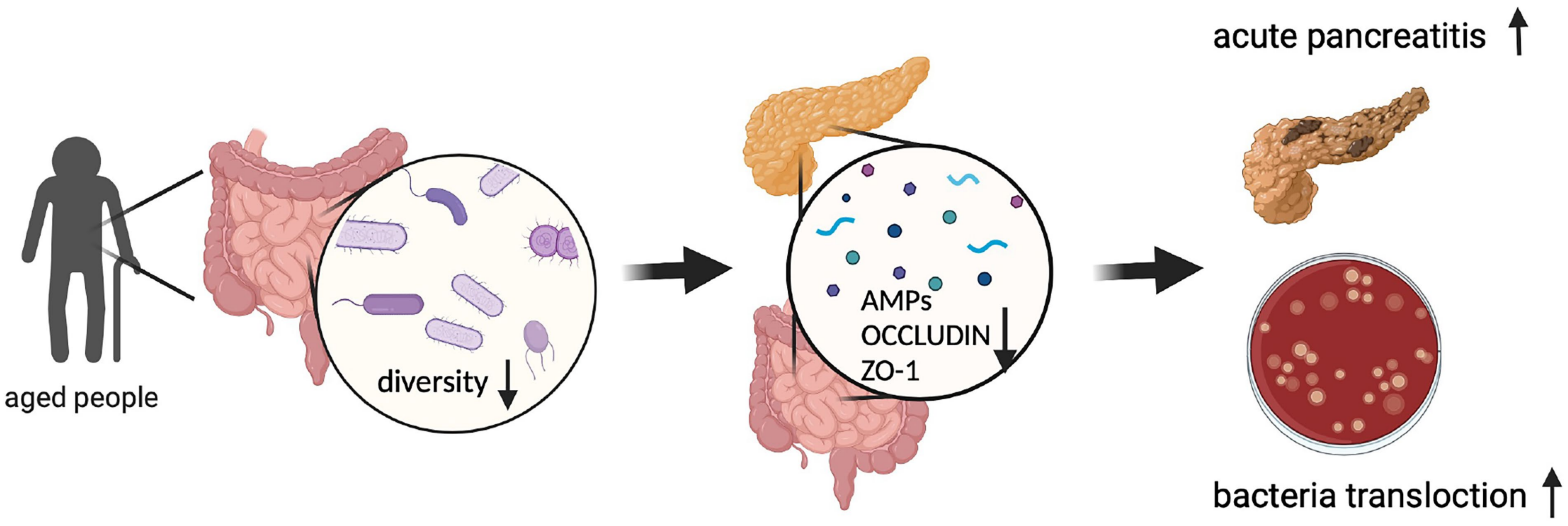
Graphic abstract. (Created with BioRender.com).

AMPs have a strong connection with the gut microbiota and a strong influence on the gut innate immune system (Ostaff et al., 2013; Brooks et al., 2021; Sun et al., 2021). Ahuja et al. find that pancreatic antimicrobial peptides CRAMP is vital for the gut innate immunity (Ahuja et al., 2017). Huang et al. find that protein expression of lysozyme in ileum decreased significantly in the hypertriglyceridemia-related acute necrotizing pancreatitis rats (Chen et al., 2017). Brooks et al. find that the microbiota coordinates diurnal rhythms in innate immunity through several AMPs such as REG3G (Brooks et al., 2021). We detected the gene expression of three major types of AMPs in two organs. The number of AMPs decreased in the older participants. Their CRAMP levels decreased slightly in both the pancreas and ileum. LYZ1 levels decreased only in the ileum. REG3G decreased dramatically in both the pancreas and ileum, especially in the pancreas (Figures 5A,B). The heatmap of the correlation between the AMPs and the phenotype shows that the AMPs secreted from both the pancreas and ileum have negative correlations with a more severe phenotype (Figures 5C,D). The distance of AMPs in both groups of mice decreased during the recovery period (Figures 5E, 6).

### Antibiotic treatment before cerulein inducement attenuates the differences between the samples from healthy older and younger groups

**Figure 7:**
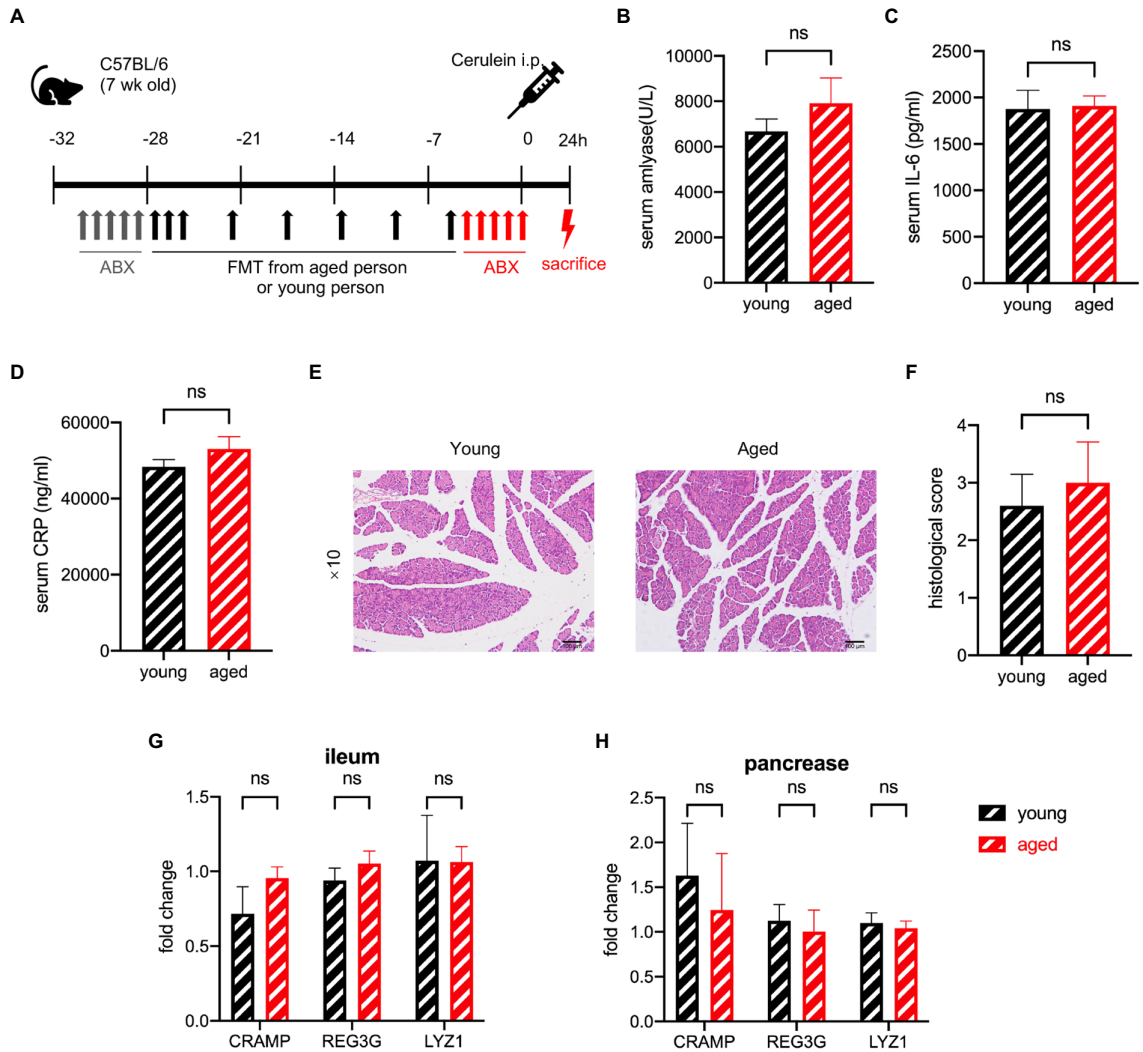
**(A)** Experimental design for gut depletion before SAP. **(B)** Serum amylase, serum IL-6 **(C)**, and serum CRP **(D)** levels are significantly increased in the old group compared to the young group. **(E)** Typical pathological image of two groups. **(F)** Histological scoring of pancreatic damage (*n* = 5 per group). Expression of AMP genes in the pancreas **(G)** and ileum **(H)** of the young group versus the old group (*n* = 7 per group). ^*^p < 0.05, ^**^p < 0.01, ^***^p < 0.001, ^****^p < 0.0001, ns, no significant difference.

To determine whether different gut microbiota irreversibly change the AMPs before AP occurs, we administered antibiotics to the mice after faecal microbiota transplantation (FMT) to remove the gut microbiota that had already been transplanted (Figure 7A). The difference in the serum levels of amylase, IL-6, and CRP disappeared with antibiotic treatment (Figures 7B–D). The histological examination showed no significant difference between the two groups (Figures 7E,F). The degree of pathological damage. in both groups was milder than that in those without antibiotic treatment, which is consistent with previous reports (Zhu et al., 2019; van den Berg et al., 2021). AMPs of the pancreas and ileum showed no difference between the two groups (Figures 7G,H). These outcomes suggest that the effect of gut microbiota works after the incidence of AP, and the differences in AMP secretion occur after disease induction.

### Antibiotic treatment after cerulein inducement shows no ability to reverse the effect of the healthy older group samples

**Figure 8:**
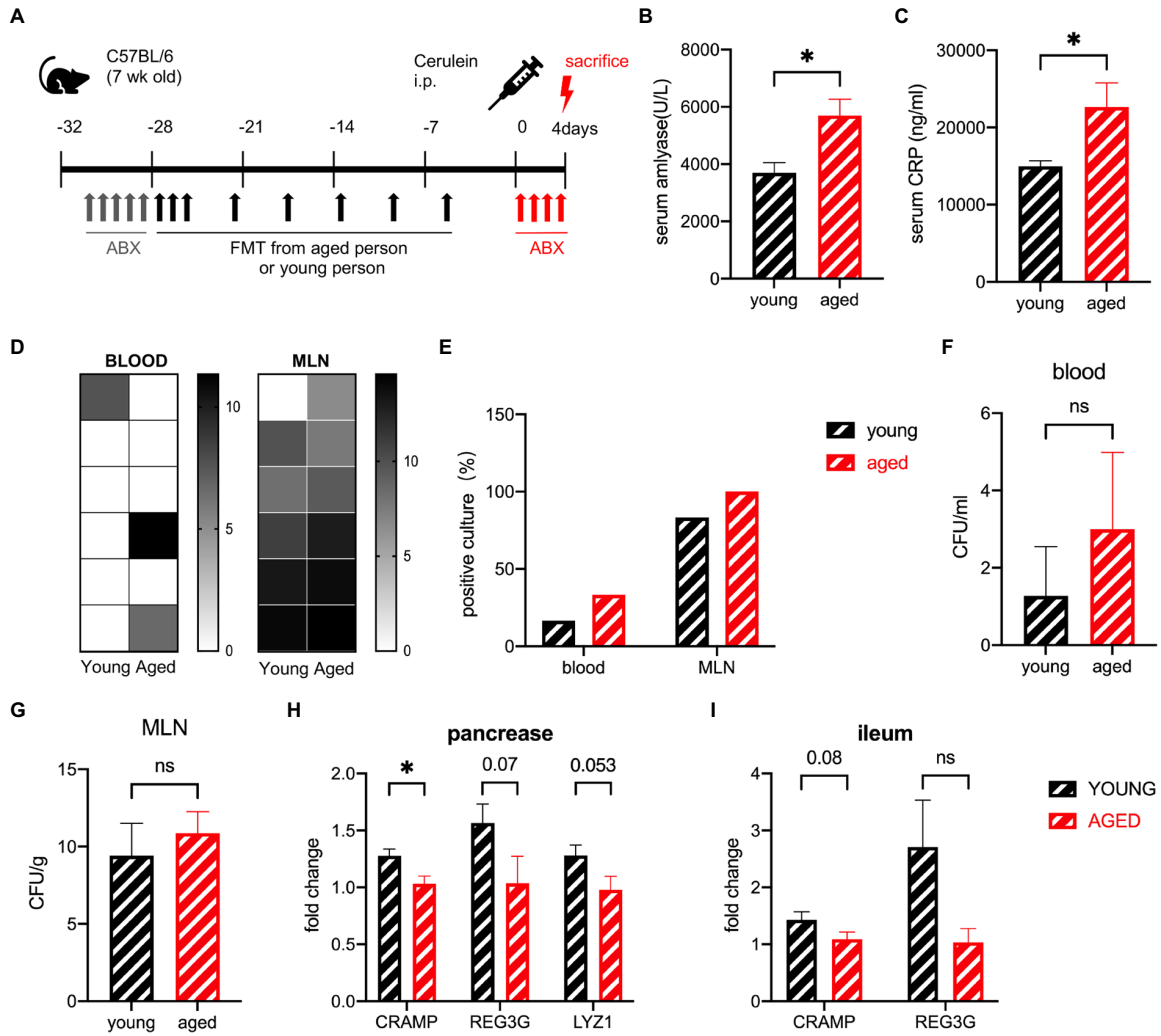
**(A)** Experimental design for gut depletion after SAP. **(B)** Serum amylase levels are significantly increased in the old group compared to the young group. **(C)** Serum CRP levels are significantly increased in the old group compared to the young group. **(D)** Heatmap of the CFU (log2) of blood and MLN. **(E)** Frequency of culture positivity in blood and MLN. CFU in the blood **(F)** and MLN **(G)**. Expression of AMP genes in the pancreas **(H)** and ileum **(I)** of the young group versus the old group (n = 6 per group). ^*^p < 0.05, ^**^p < 0.01, ^***^p < 0.001, ^****^p < 0.0001, ns, no significant difference.

To investigate whether antibiotic treatment can diminish the effect of the aged gut microbiota after SAP, the mice were treated orally with antibiotics 12 h after the first shot of cerulein (Figure 8A). The serum levels of amylase and CRP significantly increased in the old group (Figures 8B,C). Pathological examination showed that oedema and inflammatory cell infiltration had basically recovered in both groups (Supplementary Figure S4A). The percentage of positive cultures showed a slight increase in the old group, as did the CFUs (Figures 8D–G). The negative influence of aged gut microbiota was diminished by antibiotic treatment during SAP. AMPs in the pancreas and ileum were also detected. The AMPs of the old group decreased, but the distance between the two groups was narrower than before (Figures 8H,I).

## Discussion

Aging is a risk factor for AP, which has been confirmed in several clinical studies and experiments (Szakács et al., 2018; Coelho et al., 2019; Márta et al., 2019). Among aging patients with AP, the dysfunction of organs and the immune system has been reported to be the main cause of poor prognosis of AP. However, the dramatic changes in the gut microbiota of aging patients have been ignored (Lynch and Pedersen, 2016). Surprisingly, there has been a flurry of recent research on interventions by gut microbiota to ameliorate age-related diseases (Bárcena et al., 2019; Parker et al., 2022). A study on the effect of gut microbiota from the elderly people and children on osteoporosis provided a research idea for our study (Liu et al., 2021). At the same time, studies have found that the depletion of the intestinal flora caused by the Western diet can lead to a dramatic increase in the incidence of fatal bacteremia in acute pancreatitis (van den Berg et al., 2021). This suggests that the decline in gut microbiome diversity caused by aging may have a similar effect. The decreasing trends in gut microbiota diversity are strongly correlated with increasing age (O'Toole and Jeffery, 2015). Frailty (measured by the Barthel Index) has a negative correlation with species richness of the gut microbiota (Jeffery et al., 2016), which means that older people are at greater risk of gut microbiota disorders if they have diseases such as AP. Therefore, we designed this study to investigate whether the aging gut microbiota influence AP. Most studies on the gut microbiota focus on specific bacteria based on the available biological information. In this study, the bioinformatic outcome was more likely to change at the pattern level. Although the gut microbiota of older individuals is more accessible to modulate by diet than those of younger adults, they are regarded as a more chaotic environment and present a higher possibility of responding to preventive measures (Claesson et al., 2012). We found that the absolute abundance and diversity of gut microbiota in the elderly decreased dramatically, and the disappearance of non-dominant species characterized the decrease in diversity. There have been many studies on the changes in gut microbiota in aging (Claesson et al., 2012; O'Toole and Jeffery, 2015; Jeffery et al., 2016; Lynch and Pedersen, 2016; Wilmanski et al., 2021), but we found that the changes may mainly focus on the disappearance of non-dominant species.

We transplanted gut microbiota from aged and young humans into mice and observed the phenotype of acute pancreatitis in mice. Unlike most studies on acute pancreatitis, which focus on the phenotype of the disease at 24 h, we observed the manifestations of acute pancreatitis at the recovery stage. Moreover, determine the bacteremia caused by gut-derived bacterial translocation by culturing mesenteric lymph nodes and blood. Our results suggest that aging gut microbiota leads to a heavier phenotype and poorer prognosis of acute pancreatitis in mice.

AMPs can be secreted from most of the epithelium; the Paneth cells of the ileum are the main source of AMPs in the intestine (Bevins and Salzman, 2011). AMPs can also be secreted from the pancreas and contribute to approximately 10% of the proteins found in pancreatic juice.(Doyle et al., 2012) This suggests a link between the anti-infection abilities of the gut and pancreas. Also, Ahuja et al. show that the knockout of pancreatic AMPs-related genes can directly lead to fatal infection in mice (Ahuja et al., 2017). Huang et al. show that the secretion of antimicrobial peptides in ileum Panthe cells of necrotizing pancreatitis under hyperlipidemia is reduced (Huang et al., 2017). Ho et al. confirm that intestinal antimicrobial peptides have a protective effect on the body in sepsis (Ho et al., 2020). That is the reason why we are curious about AMPs of the ileum and pancreas in the context of pancreatitis. In the experiment, we found that the secretion of AMPs in the ileum and pancreas in the early stage was much higher than that in the health situation or recovery stage, so we believed that the antimicrobial peptides secreted by the pancreas and ileum might be closely related to acute pancreatitis.

In a previous study, the treatment of AP with probiotic prophylaxis did not reduce the risk of infection; furthermore, the treatment correlated with increased mortality (Besselink et al., 2008). Several studies showed that FMT aggravates AP, and antibiotic treatment positively affects AP (Zhu et al., 2019; van den Berg et al., 2021). Therefore, we focused on antibiotic treatment to determine whether it could eliminate the gap between the gut microbiota of these two age groups. The results turn out that antibiotic intervention before disease induction can narrow the difference caused by aging gut microbiota and alleviate the disease. The effects of the microbiome occur at the onset of the disease, but the hysteresis of antibiotic treatment makes it impossible to change the effects of the microbiome completely. Moreover, antibiotics such as meropenem pretreatment may lead to indirect detrimental effects during acute pancreatitis (Soares et al., 2017). The risk of antibiotics inducing multidrug-resistant pathogens has prompted the exploration of other interventions to inhibit bacterial overgrowth (Hyoju et al., 2019). This suggests that we need a better approach to the management of acute pancreatitis.

Our study had several limitations. Although we found that the transcription level of AMPs in the ileum and pancreas in the early stage was much higher than that in the health situation or recovery stage, we did not conduct further supplement or knockout experiments of these antimicrobial peptides to verify their functions. The relationship between AMPs, pancreatitis and gut microbiota remains only a phenomenon. Furthermore, we want to deepen the work directly in the model of acute pancreatitis rather than under the aging influence. Secondly, we failed to verify the inhibitory effect of pancreatic and ileum antimicrobial peptides on bacteria more directly. Finally, there are various types of antimicrobial peptides, but we only selected three representative ones for detection. And due to the strong specificity of antimicrobial peptides on bacteria, these may not fully explain the difference between groups of bacterial translocation.

In summary, the results of this study provide evidence that aging can affect the severity and prognosis of AP through gut microbiota, and this phenomenon may be caused by the secretion of AMPs stimulated by gut microbiota. Normal antibiotic treatment cannot eliminate the effects of aged gut microbiota.

## Data availability statement

The datasets presented in this study can be found in online repositories. The names of the repository/repositories and accession number(s) can be found at NCBI. BioProject number PRJNA825070.

## Ethics statement

The studies involving human participants were reviewed and approved by Ethics Committee of Minhang Hospital, Fudan University. The patients/participants provided their written informed consent to participate in this study.

## Author contributions

ZZ and LW designed this study. HJ conducted the experiments and wrote the manuscript. QC and YX collected the patient information and samples. HJ, QC, and YX contributed equally to this work. JW, XW, and JH assisted with data processing and R software. All authors contributed to the article and approved the submitted version.

## Funding

This work was supported by the Shanghai Municipal Commission of Health and Family Planning (201740238), Minhang District Science and Technology Committee (2019MHZ068), Minhang District Specific Clinics Construction Project (2020 MWTZB08) and Minhang District Health Commission Fund Project (2020FM26).

## Conflict of interest

The authors declare that the research was conducted in the absence of any commercial or financial relationships that could be construed as a potential conflict of interest.

## Publisher’s note

All claims expressed in this article are solely those of the authors and do not necessarily represent those of their affiliated organizations, or those of the publisher, the editors and the reviewers. Any product that may be evaluated in this article, or claim that may be made by its manufacturer, is not guaranteed or endorsed by the publisher.

## Supplementary material

SUPPLEMENTARY FIGURE S1 The rarefaction curve of samples.

SUPPLEMENTARY FIGURE S2 **(A)** The 16SrDNA general PCR results of mice stool samples after antibiotic treatment. **(B)** The FMT homology analysis by SourceTracker. **(C)** The typical plate images of abx treatment.

SUPPLEMENTARY FIGURE S3 **(A)** Anatomical images in mice in the young group on day 4. **(B)** Typical pathological image of two groups on day 4.

SUPPLEMENTARY FIGURE S4 **(A)** Typical pathological image of two groups had antibiotic treatment after disease induction.
