# Supplementary figures and images for: Effect of aging on acute pancreatitis through gut microbiota
Source: Front Microbiol. 2022 Jul 28;13:897992. doi: 10.3389/fmicb.2022.897992 (PMC9366017; doi:10.3389/fmicb.2022.897992)

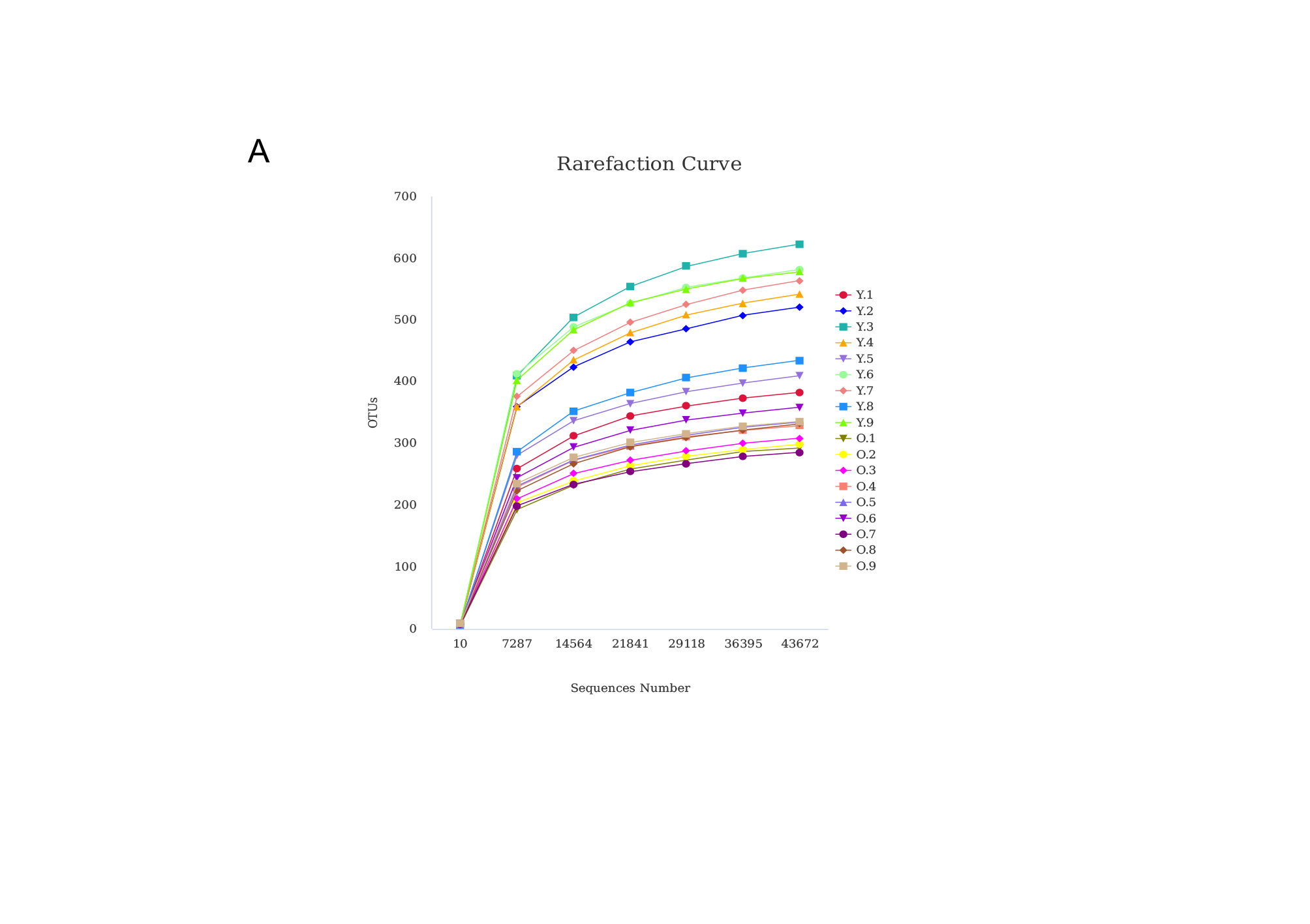

Supplement: SUPPLEMENTARY FIGURE S1 — The rarefaction curve of samples. [file Image_1.TIF]

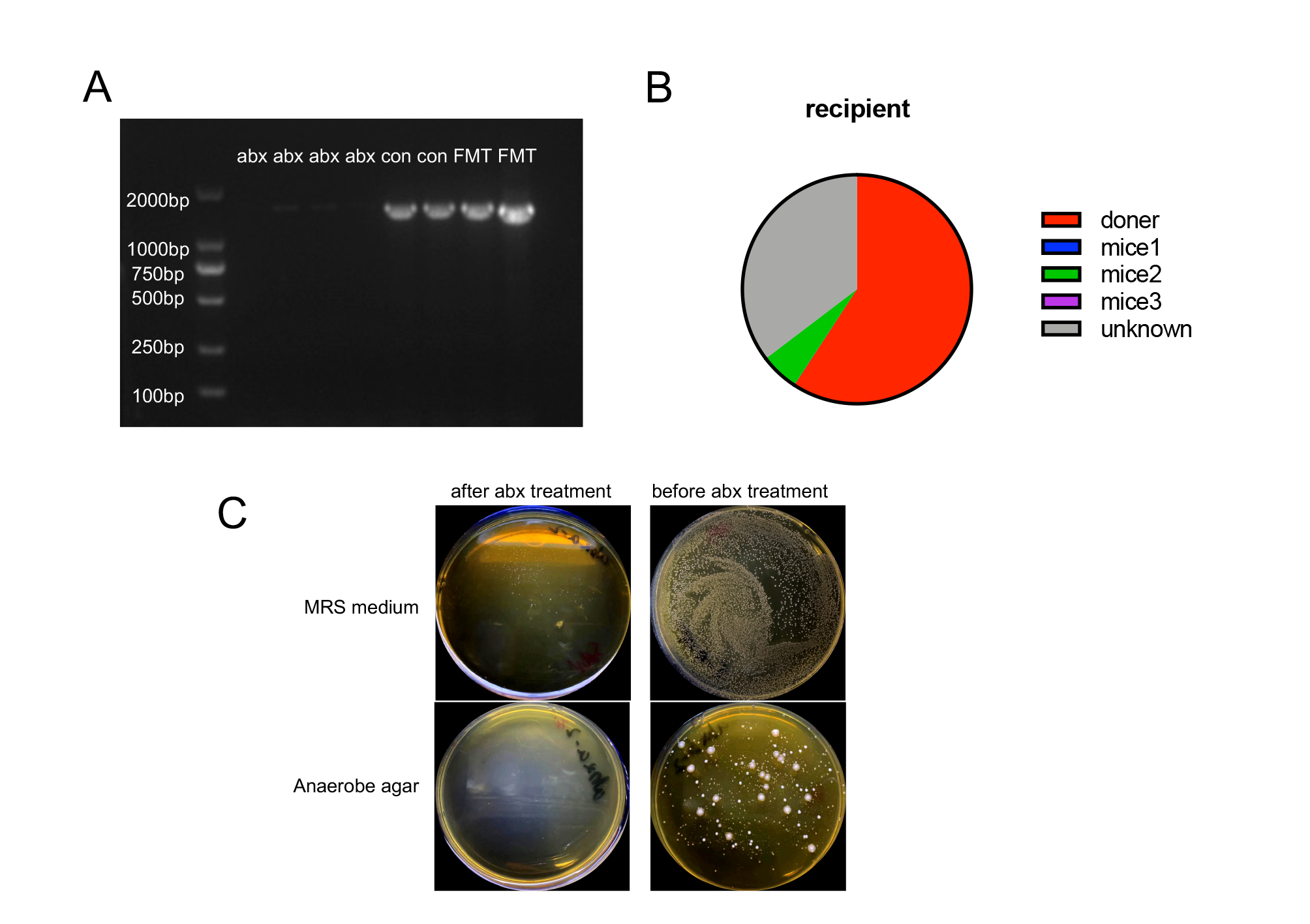

Supplement: SUPPLEMENTARY FIGURE S2 — (A) The 16SrDNA general PCR results of mice stool samples after antibiotic treatment. (B) The FMT homology analysis by SourceTracker. (C) The typical plate images of abx treatment. [file Image_2.TIF]

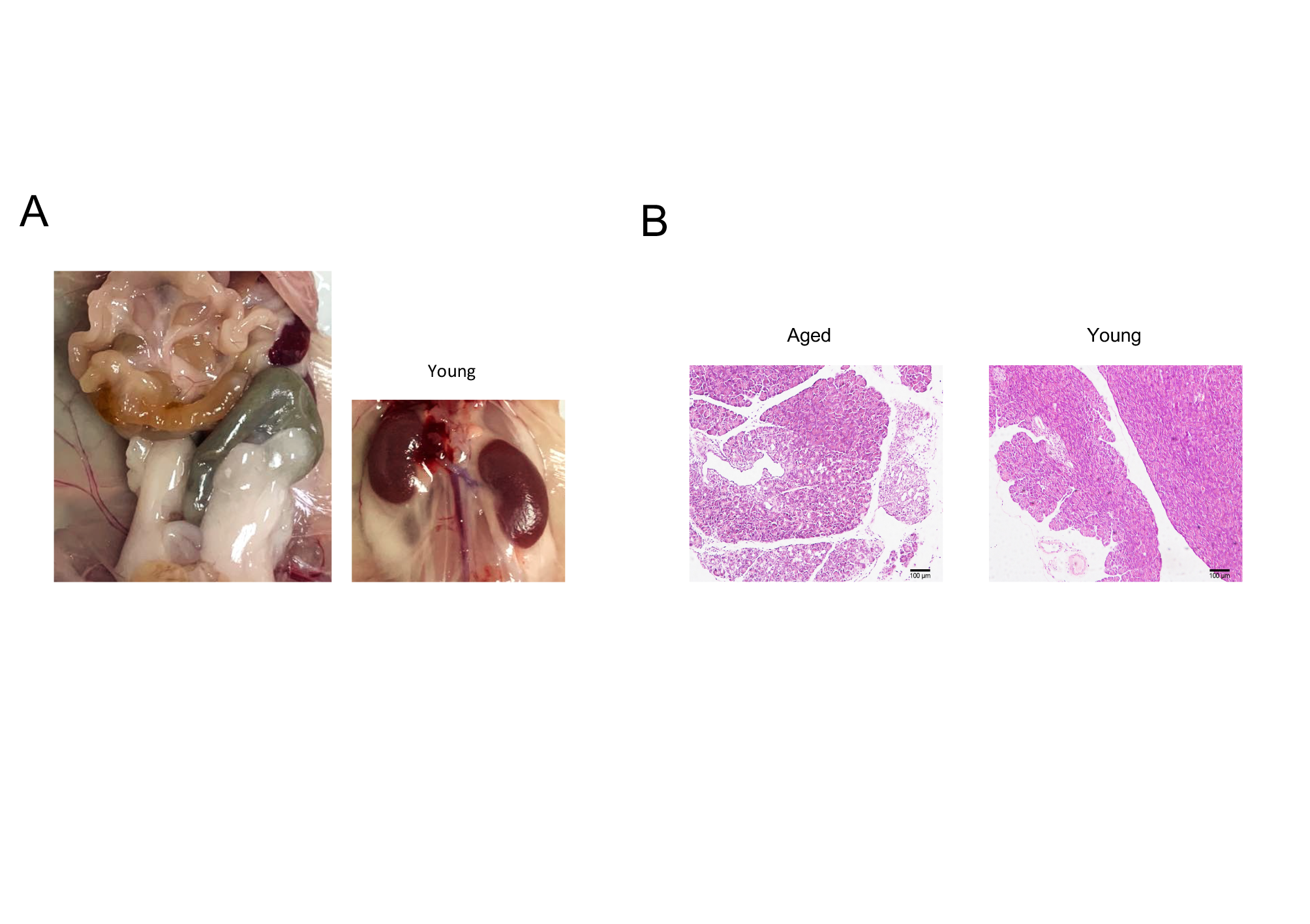

Supplement: SUPPLEMENTARY FIGURE S3 — (A) Anatomical images in mice in the young group on day 4. (B) Typical pathological image of two groups on day 4. [file Image_3.TIF]

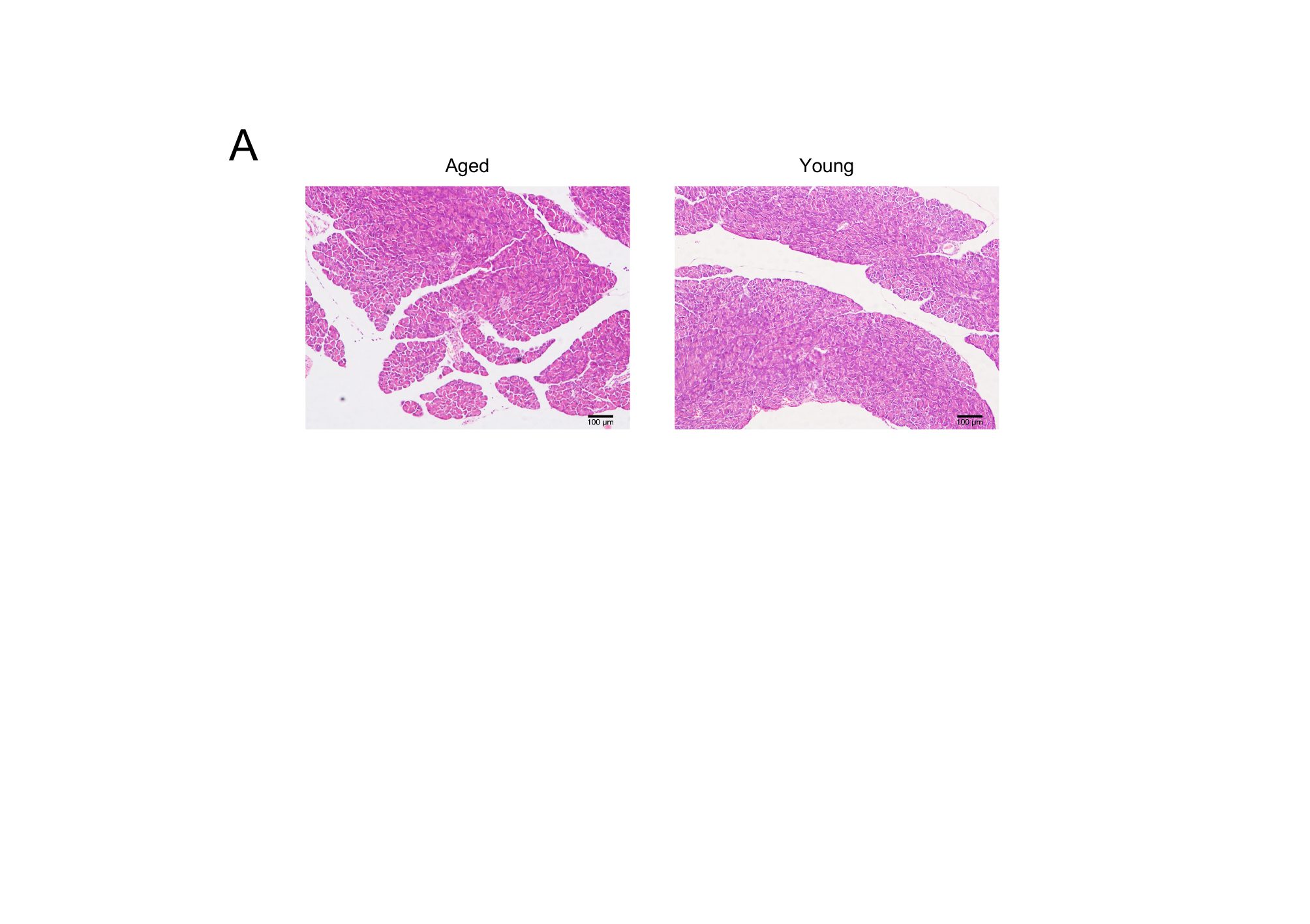

Supplement: SUPPLEMENTARY FIGURE S4 — (A) Typical pathological image of two groups had antibiotic treatment after disease induction. [file Image_4.TIF]
