# Supplementary material for: Effect of aging on acute pancreatitis through gut microbiota
Source: Front Microbiol. 2022 Jul 28;13:897992. doi: 10.3389/fmicb.2022.897992 (PMC9366017; doi:10.3389/fmicb.2022.897992)
Supplement: Supplementary file 5 [file Data_Sheet_1.PDF]

editage

Editing Certificate

This document certifies that the manuscript listed below has been edited to ensure language and grammar accuracy and is error free in these aspects. The logical presentation of ideas and the structure of the paper were also checked during the editing process. The edit was performed by professional editors at Editage, a division of Cactus Communications. The sections titled references were not edited by Editage upon the author's request.

The author's core research ideas were not altered during the editing process. Editage guarantees the quality of editing with the assumption that our suggested changes have been accepted and the edited text has not been altered without the knowledge of our editors.

MANUSCRIPT TITLE

Aging affects acute pancreatitis through gut microbiota by regulating antimicrobial peptides

AUTHORS

Hui Jing

ISSUED ON

March 03, 2022

JOB CODE

EUHJU_1

THE EDITAGE Promise QUALITY GUARANTEE

Vikas Narang

Vikas Narang
Chief Operating Officer - Editage

editage

CACTUS

Editage, a brand of Cactus Communications, offers professional English language editing and publication support services to authors engaged in over 1300 areas of research. Through its community of experienced editors, which includes doctors, engineers, published scientists, and researchers with peer review experience, Editage has successfully helped authors get published in internationally reputed journals. Authors who work with Editage are guaranteed excellent language quality and timely delivery.

impact.science

researcher.life

lifesciences.cactusglobal.com

GLOBAL :
+1(833) 979-0061 | request@editage.com

CHINA :
400-120-3020 | fabiao@editage.cn
