# Supplementary material for: Effect of aging on acute pancreatitis through gut microbiota
Source: Front Microbiol. 2022 Jul 28;13:897992. doi: 10.3389/fmicb.2022.897992 (PMC9366017; doi:10.3389/fmicb.2022.897992)
Supplement: Supplementary file 6 [file Table_1.DOCX]

Table S1. The primers in the study

| GAPDH  (glyceraldehyde-3-phosphate dehydrogenase) | F | GGTGAAGGTCGGTGTGAACG |
| --- | --- | --- |
|  | R | CTCGCTCCTGGAAGATGGTG |
| OCLN  (occludin) | F | ATGTCCGGCCGATGCTCTC |
|  | R | TTTGGCTGCTCTTGGGTCTGTAT |
| TJP1  (tight junction protein 1, ZO-1) | F | TTTTTGACAGGGGGAGTGG |
|  | R | TGCTGCAGAGGTCAAAGTTCAAG |
| CAMP  (cathelicidin antimicrobial peptide) | F | GGTCACTATCACTGCTGCTGCTAC |
|  | R | GATCCAGGTCCAGGAGACGGTAG |
| REG3G  (regenerating family member 3 gamma) | F | TTCAGCGCCACTGAGCACAGAC |
|  | R | CGTGCCTATGGCTCCTATTGCT |
| LYZL1  (lysozyme like 1) | F | GTCACACTTCCTCGCTTTCC |
|  | R | TGACTGTCACCAGCATCCAT |
| 27F | F | AGAGTTTGATCCTGGCTCAG |
| 1492R | R | TACGGYTACCTTGTTACGACTT |
